# Supplementary material for: Machine learning models identify predictive features of patient mortality across dementia types
Source: Commun Med (Lond). 2024 Feb 28;4:23. doi: 10.1038/s43856-024-00437-7 (PMC10901806; doi:10.1038/s43856-024-00437-7)

Supplementary Information for "Machine Learning Models Identify Predictive Features of Patient Mortality across Dementia Types"

Jimmy Zhang[1,2]*, Luo Song[3]*, Zachary Miller[4], Kwun C. G. Chan[4], Kuan-lin Huang[1#]

* contributes equally

[1] Department of Genetics and Genomic Sciences, Center for Transformative Disease Modeling, Tisch Cancer Institute, Icahn Institute for Data Science and Genomic Technology, Icahn School of Medicine at Mount Sinai, New York, NY 10029, United States.

[2] Columbia University, New York, NY 10027, United States.

[3] School of Medicine, The University of Queensland, Herston, QLD 4006, Australia.

[4] National Alzheimer's Coordinating Center, University of Washington, Seattle, WA 98195, United States.

[#]Corresponding Author:

Kuan-lin Huang, Ph.D.
Department of Genetics and Genomic Sciences
Icahn School of Medicine at Mount Sinai
New York, NY 10029

Email: kuan-lin.huang@mssm.edu

Supplementary Table 1: Variables and their descriptors. *A selection of variable names and their corresponding descriptors according to the NACC UDS Researcher's Data Dictionary.*

Variable Name	Descriptor
ANIMALS	Animals — Total number of animals named in 60 seconds
BPDIAS	Subject blood pressure (sitting), diastolic
CDRGLOB	Global CDR
CDRLANG	Language
CDRSUM	Standard CDR sum of boxes
COGVIS	Indicate whether the subject currently is meaningfully impaired, relative to previously attained abilities, in visuospatial function
COMMUN	Community affairs
CVCHF	Congestive heart failure
EDUC	Years of education
ENERGY	Do you feel full of energy?
HEIGHT	Subject's height (inches)
HISPANIC	Hispanic/Latino ethnicity
HOMEHOBB	Home and hobbies
HRATE	Subject resting heart rate (pulse)
HYPERCHO	Hypercholesterolemia
HYPERTEN	Hypertension
INCONTU	Incontinence — urinary
INDEPEND	Level of independence
MMSELAN	Language of MMSE administration
MOMODE	Mode of onset of motor symptoms
MOSLOW	Indicate whether the subject currently has meaningful changes in motor function — Slowness
NACCADC	ADC at which subject was seen
NACCAGE	Subject's age at visit
NACCBMI	Body mass index (BMI)

NACCETPR	Primary etiologic diagnosis (MCI; impaired, not MCI; or dementia)
NACCGDS	Total GDS Score
NACCMMSE	Total MMSE score (using D-L-R-O-W)
NACCMOTF	Indicate the predominant symptom that was first recognized as a decline in the subject's motor function
NACCNIHR	Derived NIH race definitions
ORIENT	Orientation
PERSCARE	Personal care
RESIDENC	Type of residence
SEX	Subject's sex
SHOPPING	In the past four weeks, did the subject have any difficulty or need help with: Shopping alone for clothes, household necessities, or groceries
SMOKYRS	Total years smoked cigarettes
STOVE	In the past four weeks, did the subject have any difficulty or need help with: Heating water, making a cup of coffee, turning off the stove
TOBAC100	Smoked more than 100 cigarettes in life
TRAILA	Trail Making Test Part A — Total number of seconds to complete
TRAILB	Trail Making Test Part B — Total number of seconds to complete
TRAILBRR	Part B — Number of commission errors
TRAVEL	In the past four weeks, did the subject have any difficulty or need help with: Traveling out of the neighborhood, driving, or arranging to take public transportation
VEG	Vegetables — Total number of vegetables named in 60 seconds
WEIGHT	Subject's weight (lbs)

Supplementary Table 2: List of final hyperparameters. *Hyperparameters used in (A) the two-feature models (age + standard global CDR), (B) the multi-factorial models, and (C) the dementia type models. All hyperparameters were selected through fifty rounds of Bayesian optimization with five-fold cross validation, with the exception of the 'scale_pos_weight' parameter, which was manually set to the ratio between the number of samples in the negative class (survival) and the number of samples in the positive class (mortality) for the given dataset. All other unspecified values were initialized to the default values of the XGBoost algorithm.*

A)

Hyperparameter	One-Year Survival Model	Three-Year Survival Model	Five-Year Survival Model	Ten-Year Survival Model
**Number of trees (n_estimators)**	211	214	146	234
**Maximum depth (max_depth)**	2	2	2	2
**Learning rate (learning_rate)**	0.2	0.143	0.030	0.106
**Percentage of features used per tree (colsample_bytree)**	1.0	0.796	0.913	1.0
**Percentage of samples used per tree (subsample)**	1.0	0.344	0.377	0.2
**Minimum sum of instance weight needed per child (min_child_weight)**	25	17	16	8
**Gamma regularization parameter (gamma)**	0.01	1.623	0.596	0.01
**Balance between positive and negative weights (scale_pos_weight)**	15.081	3.862	1.872	0.476

B)

Hyperparameter	One-Year Survival Model	Three-Year Survival Model	Five-Year Survival Model	Ten-Year Survival Model
**Number of trees (n_estimators)**	101	226	264	299
**Maximum depth (max_depth)**	3	5	9	8
**Learning rate (learning_rate)**	0.019	0.022	0.012	0.046
**Percentage of features used per tree (colsample_bytree)**	0.470	0.824	0.815	0.619
**Percentage of samples used per tree (subsample)**	0.325	0.865	0.764	0.665

**Minimum sum of instance weight needed per child (min_child_weight)**	11	20	20	7
**Gamma regularization parameter (gamma)**	3.492	0.398	1.947	1.075
**Balance between positive and negative weights (scale_pos_weight)**	15.081	3.862	1.872	0.476

C)

Hyperparameter	No Dementia	AD	Unknown	FTLD	LBD	VaD	Other	Dep
**Number of trees (n_estimators)**	78	107	214	148	141	218	126	146
**Maximum depth (max_depth)**	10	7	3	10	10	10	2	8
**Learning rate (learning_rate)**	0.043	0.065	0.054	0.01	0.01	0.01	0.100	0.019
**Percentage of features used per tree (colsample_bytree)**	0.219	0.561	0.650	0.2	0.2	1.0	0.218	0.735
**Percentage of samples used per tree (subsample)**	0.968	0.914	0.838	1.0	1.0	1.0	0.522	0.538
**Minimum sum of instance weight needed per child (min_child_weight)**	24	9	13	8	25	22	15	16
**Gamma regularization parameter (gamma)**	1.009	0.163	1.640	0.01	0.01	0.01	0.751	5.691
**Balance between positive and negative weights (scale_pos_weight)**	8.609	1.748	6.715	1.061	1.038	2.05	3.862	10.4

Supplementary Table 3: Characteristics of data entries separated by cognitive status

Characteristic	Dementia severity			
	**Normal cognition**	**Impaired-not-MCI**	**MCI**	**Dementia**
Number of Entries	76502	7302	28385	51593
Age (years old), mean (SD)	74 (10)	74 (10)	76 (10)	76 (11)
Female, n (%)	49944 (65.3%)	4123 (56.5%)	14475 (51.0%)	26221 (50.8%)
Education (years), mean (SD)	16 (6)	15 (5)	16 (6)	15 (8)
Race, n (%)				
White	62443 (81.6%)	5654 (77.4%)	22745 (80.1%)	44099 (85.5%)
Black/African American	11005 (14.4%)	1200 (16.4%)	4164 (14.7%)	4742 (9.2%)
American Indian/Alaskan Native	418 (0.5%)	43 (0.6%)	180 (0.6%)	339 (0.7%)
Native Hawaiian/Pacific Islander	44 (0.1%)	7 (0.1%)	15 (0.1%)	64 (0.1%)
Asian	1939 (2.5%)	163 (2.2%)	812 (2.9%)	1054 (2.0%)
Other/multiracial/unknown	653 (0.9%)	235 (3.2%)	469 (1.7%)	1295 (2.5%)
Hispanic ethnicity, n (%)	4191 (5.5%)	711 (9.7%)	2161 (7.6%)	3975 (7.7%)
>= 1 APOE e4 allele, n (%)	20236 (29.6%)	1894 (25.9%)	9473 (33.4%)	22427 (43.5%)

Supplementary Table 4: Predictive performance of the two-feature models (age + standard global CDR)

Survival-time Threshold	Internal Test Set			External Test Set		
	**Accuracy (95% CI)**	**AUC-ROC (95% CI)**	**AUC-PR (95% CI)**	**Accuracy (95% CI)**	**AUC-ROC (95% CI)**	**AUC-PR (95% CI)**
One-year	0.747 (0.733-0.771)	0.804 (0.799-0.824)	0.240 (0.215-0.264)	0.785	0.853	0.277
Three-year	0.744 (0.730-0.758)	0.787 (0.778-0.795)	0.494 (0.475-0.518)	0.777	0.808	0.482
Five-year	0.720 (0.709-0.730)	0.774 (0.764-0.781)	0.643 (0.627-0.658)	0.732	0.772	0.577
Ten-year	0.701 (0.672-0.705)	0.768 (0.750-0.770)	0.865 (0.854-0.875)	0.656	0.714	0.636

Supplementary Figure 1: Survival analysis of other diseases by standard global CDR. *Kaplan-Meier estimator curves separated by standard global CDR and stratified by disease.*

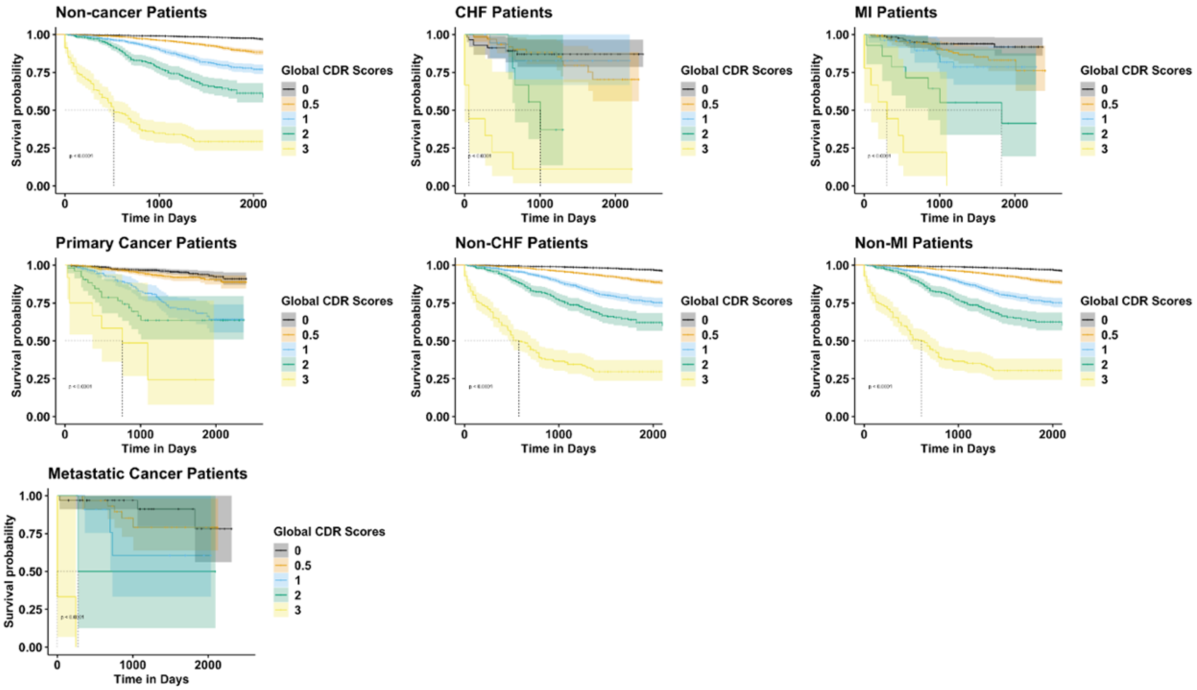

Supplementary Figure 2: Flowchart of data splitting. *The diagram illustrates how the train/internal test set and external test set were split based on the setting of survival year.*

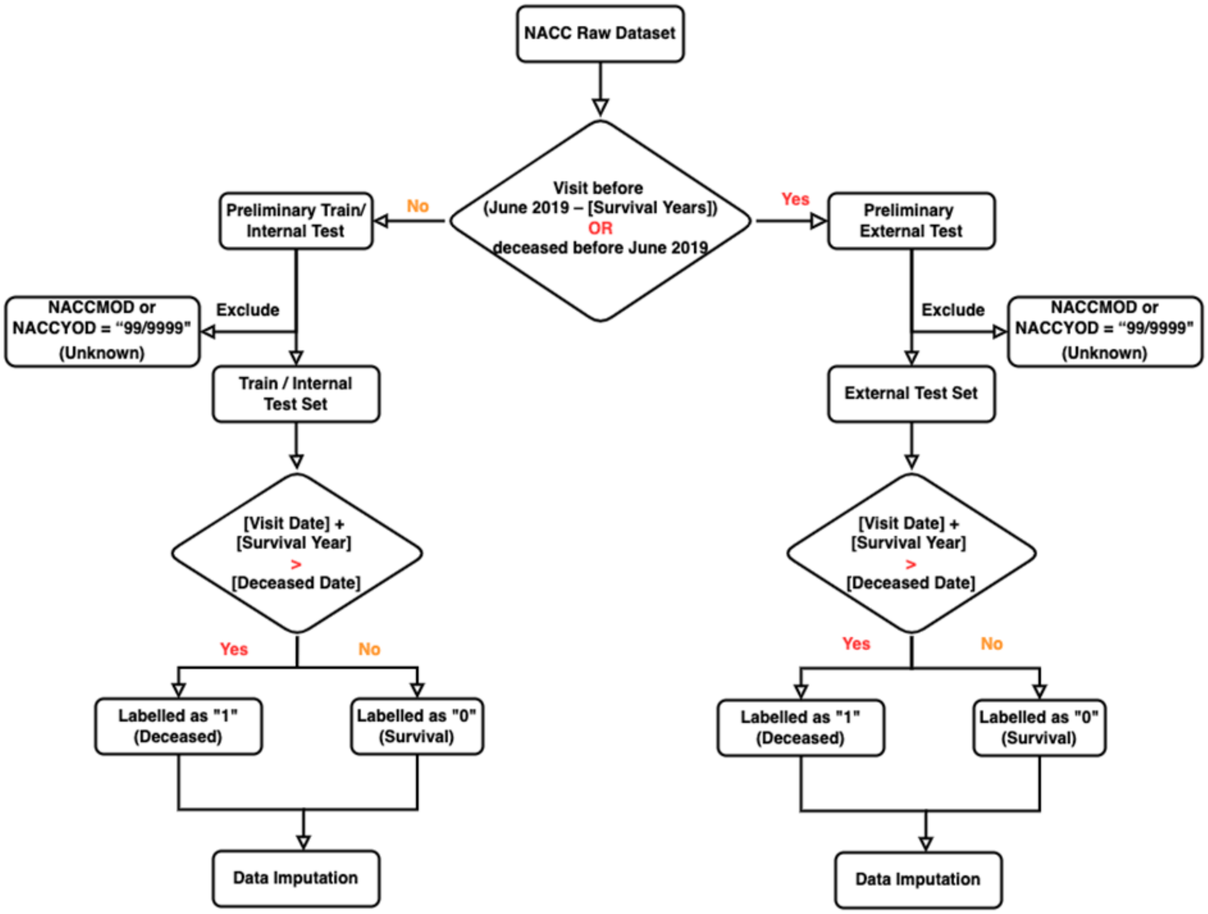

Supplementary Figure 3: Receiver operating characteristic curves of the two-feature models (age + standard global CDR). *Receiver operating characteristic curves of (A) the one-year survival model, (B) the three-year survival model, (C) the five-year survival model, and (D) the ten-year survival model.*

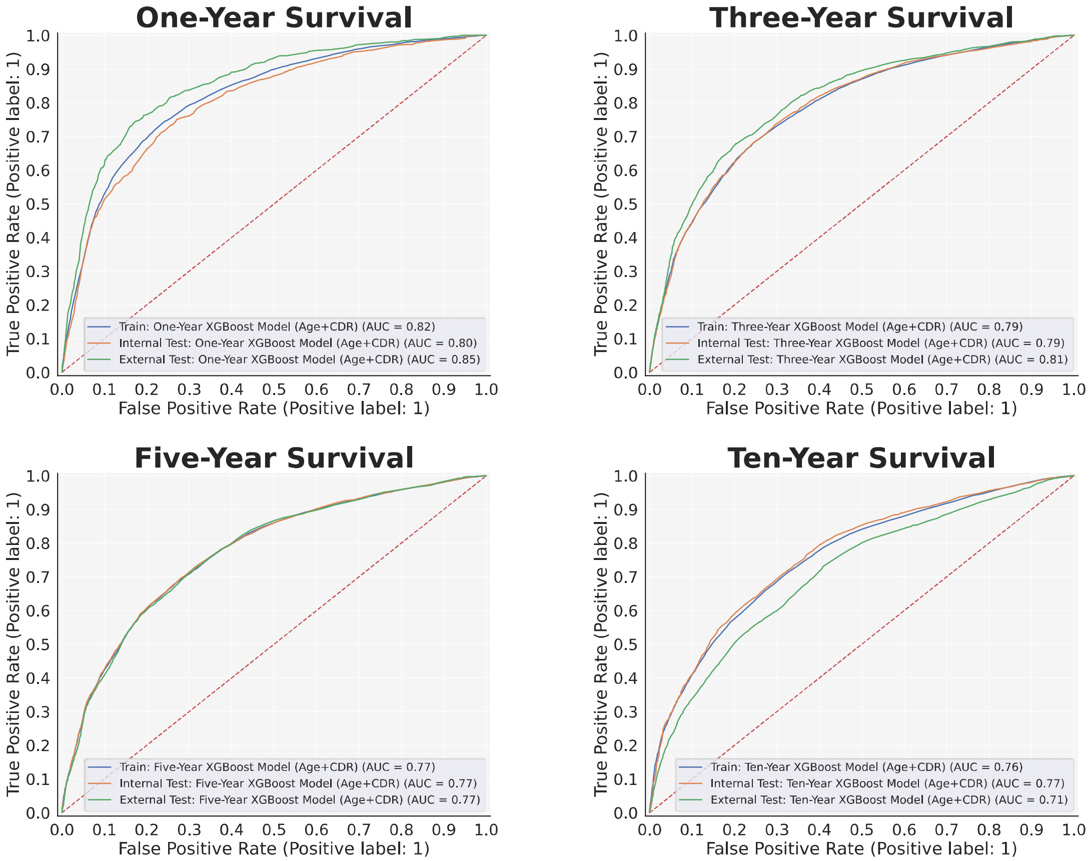

Supplementary Figure 4: Feature selection. *(A) SHapley Additive exPlanations (SHAP) bar plots of the one-year, three-year, five-year, and ten-year survival models in the training set. Features are ranked by mean absolute SHAP value. The top five features of each of the four models are indicated by the red line. (B) Line plot of the total number of features to be included for various cutoffs for the top (n) features selected from each survival threshold. (C) Venn diagram of the number of overlapping features among the top five features from each survival threshold.*

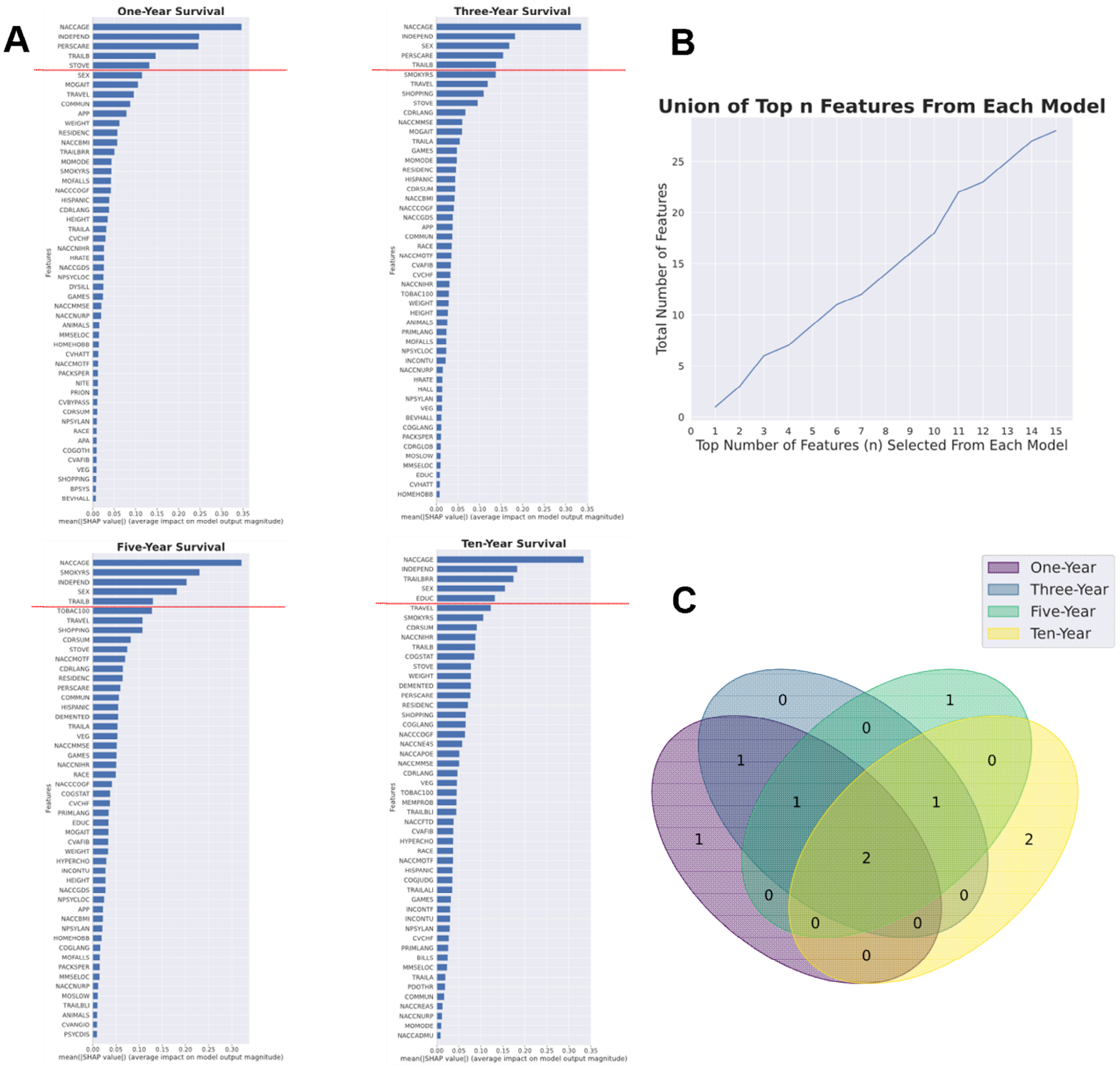

Supplementary Figure 5: Receiver operating characteristic curves of the multi-factorial models separated by Alzheimer's disease center. *Receiver operating characteristic curves stratified by Alzheimer's disease center of (A) the one-year survival model, (B) the three-year survival model, (C) the five-year survival model, and (D) the ten-year survival model. Performance within each site was evaluated using leave-one-(site)-out cross-validation (LOOCV), in which training data from all but one site was used for training, while the internal test data from the leftover site was used for testing. The code corresponding to each Alzheimer's disease center (ADC) is a randomly generated ID that replaces the actual ADC ID.*

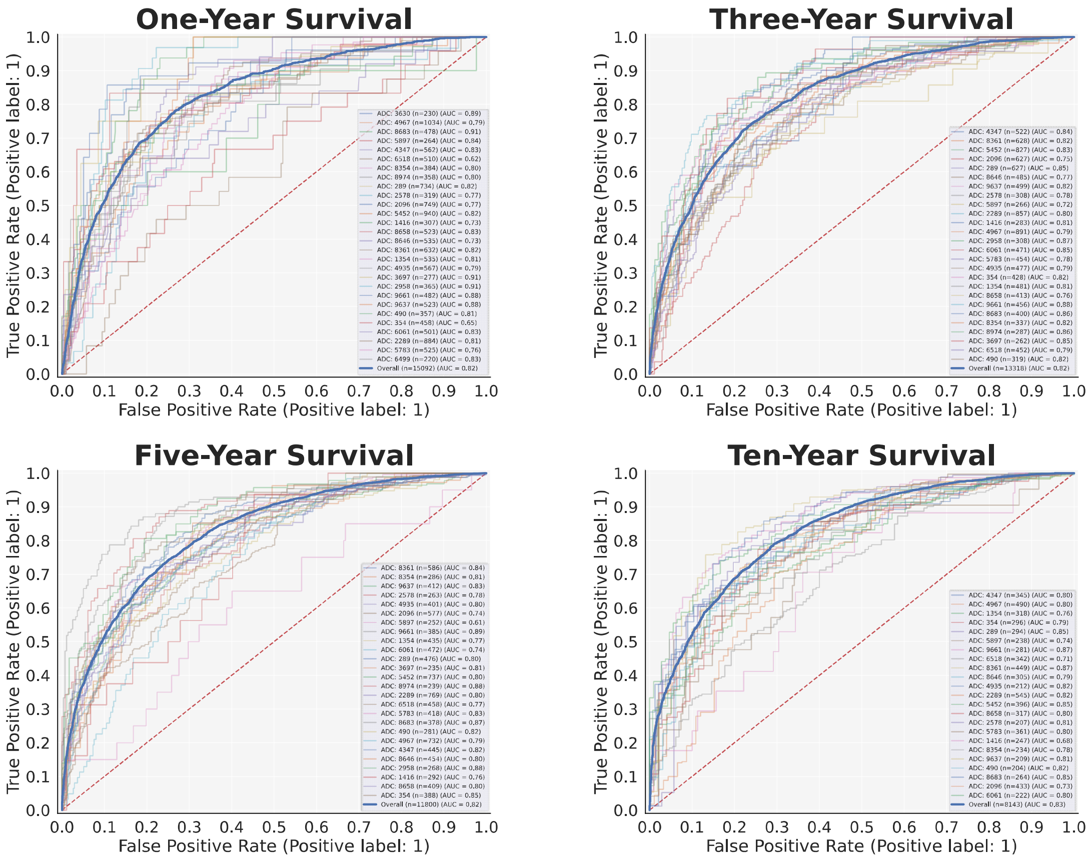

Supplement: Supplementary file 2 — Supplemental Materials [file 43856_2024_437_MOESM2_ESM.pdf]
